# Non-high-density lipoprotein cholesterol to high-density lipoprotein cholesterol ratio in relation to benign prostatic hyperplasia: A cross-sectional study

**DOI:** 10.1097/MD.0000000000042840

**Published:** 2025-06-13

**Authors:** Zhimin Lu, Honglei Wan, Wenzhan Liu, Jiantong Zhao, Mingtao Guo

**Affiliations:** a Department of Urology, Handan First Hospital, Handan, Hebei Province, China.

**Keywords:** BPH, cross-sectional study, lipid ratio, NHANES, NHHR

## Abstract

The relationship between non-high-density lipoprotein cholesterol to high-density lipoprotein cholesterol (NHHR) and benign prostatic hyperplasia (BPH) is controversial and has rarely been studied in the U.S. population. This study utilized data from 4 cycles of the National Health and Nutrition Examination Survey conducted between 2001 and 2008, and the diagnosis of BPH relied primarily on self-reporting by participants. In order to deeply analyze the association between the NHHR and BPH, the study adopted a variety of statistical tools including multivariate logistic regression, smoothed curve fitting, and subgroup analysis. Together, these methods were designed to reveal potential associations between the 2 . A total of 5687 participants were enrolled in this study, including 873 BPH participants and 4814 controls. The NHHR was generally lower in the BPH group than in the control group. After fully adjusting for all confounders, the risk of developing BPH was reduced by 8% (95% CI = 0.85–0.96, *P* = .002) for each unit increase in NHHR. Compared with the first quartile of NHHR, those in the fourth quartile of NHHR were 24% less likely to develop BPH (95% CI = 0.60–0.96, *P* = .024). Subgroup analyses found that NHHR was negatively associated with BPH in those aged 60 years or older, those who were married or living with a partner, those with a college degree or higher, former smokers, nondrinkers, and those with a BMI < 25. NHHR may have a potential protective effect on BPH. However, its exact biological mechanism remains to be elucidated by further scientific studies.

## 1. Introduction

Benign prostatic hyperplasia (BPH), is a noncancerous enlargement of the prostate gland that occurs as a result of the overgrowth of epithelial and fibromuscular cells in the transitional zone and its surrounding urethral region. This growth is disorganized and results in an increase in the size of the prostate.^[1]^ In older men, BPH is strongly associated with the development of lower urinary tract symptoms (LUTS). These symptoms cover a range of problems such as urgency to urinate, frequent nocturnal urination, frequent urination, discomfort during urination, incomplete emptying of the bladder, difficulty in urinating, and diminished or intermittent interruptions in urine flow.^[2]^ BPH may cause pressure on the urethra, which in turn increases resistance to urine flow, a condition known as bladder outlet obstruction (BOO). The presence of BOO may not only alter the function of the bladder, such as causing overactivity of the urethral muscles or diminishing their ability to contract, but may also manifest in LUTS, urinary tract infections, or urinary retention, among other problems. BPH and BOO pose a significant health challenge and medical and financial burden to older men. The incidence and prevalence of BPH and LUTS are rising rapidly, in line with the global trend of population aging.^[3]^ The pathogenesis of BPH involves a variety of factors, including changes in sex hormone levels, the action of neurotransmitters, inflammatory responses, dietary habits, and changes in the microbial environment.^[4]^

Current research suggests that metabolic syndrome (MS) and it is accompanying comorbid symptoms, such as changes in sex hormone levels and mild inflammatory responses, are strongly associated with BPH. These factors may play a key role in the development of BPH.^[5]^ The risk of BPH has been associated with abnormal lipid levels in various ethnic groups.^[6]^ Elevated levels of low-density lipoprotein cholesterol (LDL) commonly cause many diseases, especially cardiovascular disease (CVD).^[7]^ However, the presence of LDL is not solely detrimental. For healthy men at baseline, increases in adiposity and serum LDL-C levels may even reduce their risk of developing BPH over the next 5 years.^[8]^ NHHR is an emerging lipid marker used to assess the risk of atherosclerosis. NHHR may have greater diagnostic efficacy as a predictor of insulin resistance and potential risk of MS than traditional lipid markers.^[9]^ Recent studies have pointed out that NHHR can be used as an independent indicator to predict the risk of developing chronic kidney disease and nephrolithiasis.^[10,11]^ Although NHHR is a new indicator of lipid metabolism, its potential association with BPH has not been fully investigated. The present study, based on the NHANES dataset from 2001 to 2008, aims to explore the possible association between NHHR levels and BPH incidence. Through this exploration, we hope to uncover new avenues for future research in the relationship between lipid metabolism and BPH.

## 2. Methods

### 2.1. Data source

National Health and Nutrition Examination Survey (NHANES) is a comprehensive survey designed to collect health, nutrition, and socioeconomic data from diverse ethnic groups and individuals with varying health statuses in the United States. The program is committed to conducting comprehensive health and nutritional evaluations of participants every 2 years, and the process is overseen and approved by the Ethics Committee of the National Center for Health Statistics. The database covers the results of questionnaires, physical examinations, and laboratory tests. These data are publicly accessible through the official NHANES website.

### 2.2. Research population

This study utilized data from 4 cycles of the NHANES survey conducted between 2001 and 2008, focusing on the correlation between NHHR and BPH. Initially, the study population consisted of 14,658 participants. After excluding 35,771 individuals without BPH data and 200 individuals with missing NHHR data, a total of 5687 participants met the study criteria. No additional review was required for reasons that the official NHANES website provided all the necessary data and all participants had signed informed consent and passed the necessary ethical reviews.

### 2.3. Outcome variable

The presence of prostate enlargement was considered as a key outcome variable in this study. This variable was based on data collected in the NHANES Health Questionnaire and relied heavily on participants’ responses to questions about prostate status (KIQ_P) during a face-to-face interview. The first question was, “Has your doctor ever told you that you have an enlarged prostate?” If the participant answered “no,” they were not considered to have BPH; if they answered “yes,” they were further asked the second question, “Is this enlargement benign? “ Participants were included as BPH patients only if they answered “yes” to this question. Cases with cancerous enlargement of the prostate or missing data were excluded from the study.^[12]^

### 2.4. Exposure variable

In this investigation, we used NHHR as the primary exposure variable, which measures the ratio of non-high-density lipoprotein cholesterol (non-HDL-C) to high-density lipoprotein cholesterol (HDL-C).^[13]^ To calculate non-HDL-C, we subtracted the value of HDL-C from the individual’s total cholesterol (TC). Based on the subjects’ NHHR values, we categorized them into 4 different classes to simplify the subsequent data analysis process.

### 2.5. Covariates

Based on previous study, the following factors were selected as covariates to be considered: age, race, marital status, education, poverty-to-income ratio (PIR), smoking status, drinking habits, body mass index (BMI), diabetes, and hypertension. Marital status was categorized as married/cohabiting and single. Educational attainment was categorized as below high school, high school and above high school. Poverty-to-income ratio were categorized as <1.5, 1.5 to 3.5, and >3.5. Those who had never smoked more than 100 cigarettes were considered nonsmokers, while others were categorized as current smokers and former smokers based on current smoking status. Drinking habits were categorized according to the amount of alcohol consumed per day as nondrinkers, light drinkers (1–2 drinks/day), and heavy drinkers (more than 2 drinks/day), while BMI was categorized as <25, 25 to 29.9, and more than 30 kg/m², corresponding to normal weight, overweight, and obese, respectively. Participants were categorized as hypertensive if they had a mean blood pressure higher than or equal to 140/90 mm Hg or had been diagnosed with hypertension. In contrast, those with a history of diabetes or fasting blood glucose levels above 126 mg/dL were considered diabetic. Additionally, TC and high-density lipoprotein cholesterol (HDL-C) levels were included as continuous variables.

### 2.6. Statistical analysis

This study adhered to the guidelines set by the Centers for Disease Control and Prevention (CDC) to ensure the proper execution of statistical analyses. The NHHR data were categorized into 4 quartiles, with the lowest quartile (Q1) as the reference group. Categorical data were described by frequencies and percentages, and continuous variables were described by means. Multivariable logistic regression models were used to examine the relationship between NHHR and BPH incidence. Model 1 did not adjust for any confounders. Model 2 adjusted for age and race. Model 3 further adjusted for marital status, education level, PIR, smoking status, alcohol consumption, diabetes, and hypertension based on model 2. Smoothed curve fitting was utilized to explore potential nonlinear relationships between NHHR and BPH incidence. Subgroup analyses were stratified according to age, marital status, education level, PIR, smoking status, alcohol intake, diabetes, and BMI. Statistical analyses were conducted using EmpowerStats (version 2.0), R software (version 4.0.5), and SPSS 20.0 (IBM Corp., Armonk), with a *P*-value of <.05 considered statistically significant.

## 3. Results

### 3.1. Patient characteristics

**Figure 1.**
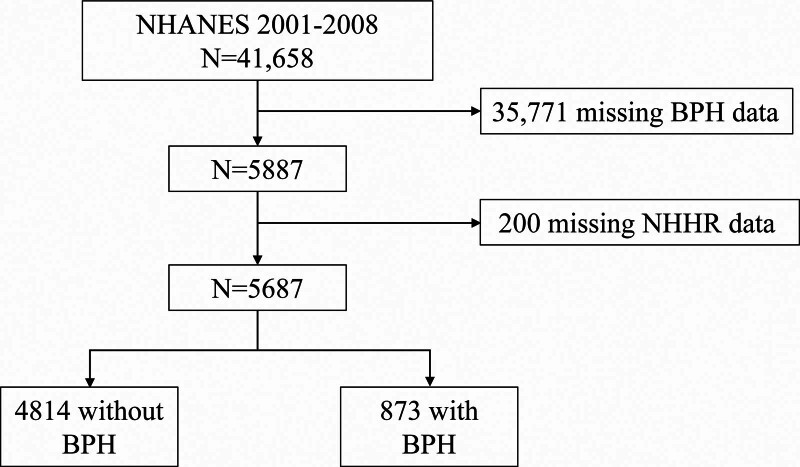
Flow chart for sample selection for the National Health and Nutrition Examination Survey, 2001 to 2008.

First, we enrolled 5687 subjects from NHANES between 2001 and 2008 based on inclusion and exclusion criteria. This included 873 BPH subjects and 4814 control subjects (Fig. 1). All participant was over 40 years old. The mean ages of the BPH and control groups were 68.42 and 59.04 years, respectively. It can be seen that the subjects in the BPH group were older. The mean NHHR values for the BPH and control groups were 3.15 and 3.49, respectively. The NHHR of the BPH group was significantly lower than that of the control group. The BPH group included a higher proportion of non-Hispanic whites, former smokers, and hypertensive patients. Additionally, the BPH group had lower TC levels (Table 1). In Table 2, baseline characteristics are described according to NHHR quartiles. The interquartile ranges of NHHR were categorized as Q1 (0.51–2.39), Q2 (2.39–3.21), Q3 (3.21–4.25), and Q4 (4.25–18.23). Patients with NHHR in the fourth quartile tended to be younger as well as belonging to the Mexican American compared to the first quartile. The prevalence of BPH in each range was 4.5%, 4.4%, 3.7%, and 2.8%, respectively. These results suggest a trend of decreasing BPH incidence as NHHR increases.

**Table 1:** Baseline characteristics of participants with or without BPH.

Characteristics	BPH		*P*-value
	No	Yes	
n	4814	873	
Age (mean + SE)	59.04 ± 0.18	68.42 ± 0.36	<.001
NHHR (mean + SE)	3.49 ± 0.02	3.15 ± 0.04	<.001
Race, n (%)			
Non-Hispanic Black	930 (16.4%)	121 (2.1%)	<.001
Non-Hispanic White	2611 (45.9%)	616 (10.8%)	
Mexican American	891 (15.7%)	85 (1.5%)	
Other Hispanic	246 (4.3%)	39 (0.7%)	
Other Race	136 (2.4%)	12 (0.2%)	
Marital status, n (%)			
Married/living with partner	3504 (61.6%)	680 (12%)	.006
Live alone	1307 (23%)	193 (3.4%)	
Missing	3 (0.1%)	0 (0%)	
Education Level, n (%)			
<High school	1561 (27.4%)	198 (3.5%)	<.001
High school	1127 (19.8%)	190 (3.3%)	
>High school	2123 (37.3%)	485 (8.5%)	
Missing	3 (0.1%)	0 (0%)	
Poverty income ratio, n (%)			
<1.5	1352 (23.8%)	180 (3.2%)	<.001
1.5–3.5	1500 (26.4%)	292 (5.1%)	
>3.5	1670 (29.4%)	355 (6.2%)	
Missing	292 (5.1%)	46 (0.8%)	
Smoking status, n (%)			
Current smoker	1164 (20.5%)	102 (1.8%)	<.001
Former smoker	1873 (32.9%)	481 (8.5%)	
No smoker	1777 (31.2%)	290 (5.1%)	
Alcohol intake, n (%)			
Light drinker	1365 (24%)	190 (3.3%)	<.001
Heavy drinker	822 (14.5%)	60 (1.1%)	
No drinker	980 (17.2%)	289 (5.1%)	
Missing	1647 (29%)	334 (5.9%)	
BMI, n (%)			
BMI < 20	112 (2%)	20 (0.4%)	.914
20 ≤ BMI < 25	1020 (17.9%)	175 (3.1%)	
25 ≤ BMI < 30	2040 (35.9%)	381 (6.7%)	
BMI ≥ 30	1536 (27%)	280 (4.9%)	
Missing	106 (1.9%)	17 (0.3%)	
Diabetes, n (%)			
No	1822 (32%)	338 (5.9%)	.275
Yes	896 (15.8%)	178 (3.1%)	
Missing	2096 (36.9%)	357 (6.3%)	
Hypertension, n (%)			
No	2253 (39.6%)	306 (5.4%)	<.001
Yes	2561 (45%)	567 (10%)	
TC, mmol/L, (mean + se)	5.19 ± 0.01	4.94 ± 0.03	<.001
HDL, mmol/L, (mean + se)	1.23 ± 0.01	1.26 ± 0.01	.082

BMI = body mass index, BPH = benign prostatic hyperplasia, HDL = high-density lipoprotein cholesterol, NHHR = non-high-density lipoprotein cholesterol to high-density lipoprotein cholesterol, SE = standard error, TC = total cholesterol.

**Table 2:** Characteristics of participants by NHHR quartile, 2001 to 2008 NHHR cycles.

Characteristics	Q1 (0.51–2.39)	Q2 (2.39–3.21)	Q3 (3.21–4.25)	Q4 (4.25–18.23)	*P*-value
n	1421	1417	1423	1426	
Age (mean ± se)	63.78 ± 0.34	61.73 ± 0.33	59.43 ± 0.34	56.99 ± 0.32	<.001
Race, n (%)					
Non-Hispanic Black	379 (6.7%)	268 (4.7%)	231 (4.1%)	173 (3%)	<.001
Mexican American	158 (2.8%)	215 (3.8%)	275 (4.8%)	328 (5.8%)	
Non-Hispanic White	801 (14.1%)	830 (14.6%)	812 (14.3%)	784 (13.8%)	
Other Race	33 (0.6%)	36 (0.6%)	30 (0.5%)	49 (0.9%)	
Other Hispanic	50 (0.9%)	68 (1.2%)	75 (1.3%)	92 (1.6%)	
Marital Status, n (%)					
Married/living with partner	965 (17%)	1060 (18.6%)	1080 (19%)	1079 (19%)	<.001
Live alone	456 (8%)	357 (6.3%)	342 (6%)	345 (6.1%)	
Missing	0 (0%)	0 (0%)	1 (0%)	2 (0%)	
Education level, n (%)					
<High school	441 (7.8%)	418 (7.4%)	440 (7.7%)	460 (8.1%)	.191
>High school	657 (11.6%)	681 (12%)	663 (11.7%)	607 (10.7%)	
High school	322 (5.7%)	317 (5.6%)	320 (5.6%)	358 (6.3%)	
Missing	1 (0%)	1 (0%)	0 (0%)	1 (0%)	
Poverty income ratio, n (%)					
<1.5	345 (6.1%)	360 (6.3%)	386 (6.8%)	441 (7.8%)	.002
1.5–3.5	483 (8.5%)	441 (7.8%)	425 (7.5%)	443 (7.8%)	
>3.5	504 (8.9%)	528 (9.3%)	535 (9.4%)	458 (8.1%)	
Missing	89 (1.6%)	88 (1.5%)	77 (1.4%)	84 (1.5%)	
Smoking status, n (%)					
Current smoker	321 (5.6%)	282 (5%)	281 (4.9%)	382 (6.7%)	<.001
Former smoker	615 (10.8%)	613 (10.8%)	573 (10.1%)	553 (9.7%)	
No smoker	485 (8.5%)	522 (9.2%)	569 (10%)	491 (8.6%)	
Alcohol intake, n (%)					
Light drinker	414 (7.3%)	400 (7%)	388 (6.8%)	353 (6.2%)	.003
Heavy drinker	221 (3.9%)	217 (3.8%)	199 (3.5%)	245 (4.3%)	
Nodrinker	335 (5.9%)	312 (5.5%)	338 (5.9%)	284 (5%)	
Missing	451 (7.9%)	488 (8.6%)	498 (8.8%)	544 (9.6%)	
BMI, n (%)					
20 ≤ BMI < 25	477 (8.4%)	314 (5.5%)	231 (4.1%)	173 (3%)	<.001
BMI < 20	86 (1.5%)	22 (0.4%)	15 (0.3%)	9 (0.2%)	
BMI ≥ 30	286 (5%)	444 (7.8%)	501 (8.8%)	585 (10.3%)	
25 ≤ BMI < 30	541 (9.5%)	604 (10.6%)	643 (11.3%)	633 (11.1%)	
Missing	31 (0.5%)	33 (0.6%)	33 (0.6%)	26 (0.5%)	
Diabetes, n (%)					
No	558 (9.8%)	561 (9.9%)	581 (10.2%)	460 (8.1%)	<.001
Missing	588 (10.3%)	594 (10.4%)	585 (10.3%)	686 (12.1%)	
Yes	275 (4.8%)	262 (4.6%)	257 (4.5%)	280 (4.9%)	
Hypertension, n (%)					
Yes	822 (14.5%)	789 (13.9%)	760 (13.4%)	757 (13.3%)	.037
No	599 (10.5%)	628 (11%)	663 (11.7%)	669 (11.8%)	
TC, mmol/L, (mean ± se)	4.48 ± 0.02	4.85 ± 0.02	5.25 ± 0.02	6.02 ± 0.03	<.001
HDL, mmol/L, (mean ± se)	1.61 ± 0.02	1.28 ± 0.01	1.12 ± 0.01	0.9 ± 0.01	<.001
NHHR, (mean ± se)	1.83 ± 0.01	2.79 ± 0.01	3.69 ± 0.01	5.43 ± 0.03	<.001
BPH, n (%)					
No	1164 (20.5%)	1167 (20.5%)	1214 (21.3%)	1269 (22.3%)	<.001
Yes	257 (4.5%)	250 (4.4%)	209 (3.7%)	157 (2.8%)	

BMI = body mass index, BPH = benign prostatic hyperplasia, HDL = high-density lipoprotein cholesterol, NHHR = non-high-density lipoprotein cholesterol to high-density lipoprotein cholesterol, SE = standard error, TC = total cholesterol.

### 3.2. Non-high-density lipoprotein cholesterol to high-density lipoprotein cholesterol and BPH

In Model 3, after fully adjusting for all confounders, the risk of BPH was reduced by 8% (95% CI = 0.85–0.96, *P* = .002) for each unit increase in NHHR. We further analyzed using NHHR subgroups (quartiles). In Model 1, the third quartile had a 22% reduction in the incidence of BPH compared with the first quartile (95% CI = 0.63–0.95, *P* = .014). The fourth quartile had a 44% lower incidence of BPH compared with the first quartile (95% CI = 0.45–0.69, *P* < .001). In Model 2, the fourth quartile had a 22% reduction in the incidence of BPH compared with the first quartile (95% CI = 0.60–0.94, *P *= .012). In Model 3, the occurrence of BPH was reduced by 24% (95% CI = 0.60–0.96, *P *= .024) in the fourth quartile compared to the first quartile. In Model 3, the p for trend test was 0.015 (Table 3).

**Table 3:** Relationship between NHHR and BPH.

Exposure	Model 1	*P*-value	Model 2	*P*-value	Model 3	*P*-value
	OR (95% CI)		OR (95% CI)		OR (95% CI)	
BPH						
NHHR	0.84 (0.79–0.89)	<.001	0.91 (0.86–0.96)	.001	0.91 (0.85–0.96)	.002
Subgroup						
Q1	1.0		1.0		1.0	
Q2	0.97 (0.80–1.17)	.758	1.03 (0.85–1.26)	.719	1.01 (0.83–1.25)	.858
Q3	0.78 (0.63–0.95)	.014	0.92 (0.75–1.13)	.452	0.89 (0.71–1.10)	.297
Q4	0.56 (0.45–0.69)	<.001	0.75 (0.60–0.94)	.015	0.76 (0.60–0.96)	.024
*P* for trend		<.001		.012		.015

Model 1: No variable adjustment was included, Model 2: adjusted for age and race, Model 3: Model 2 and adjusted for marital status, education level, PIR, smoking status, alcohol intake, body mass index (BMI), diabetes, and hypertension.

95% CI = 95% confidence interval, BPH = benign prostatic hyperplasia, NHHR = non-high-density lipoprotein cholesterol to high-density lipoprotein cholesterol, PIR = poverty-to-income ratio, OR = odds ratio.

### 3.3. Nonlinear relationship between NHHR and BPH

**Figure 2.**
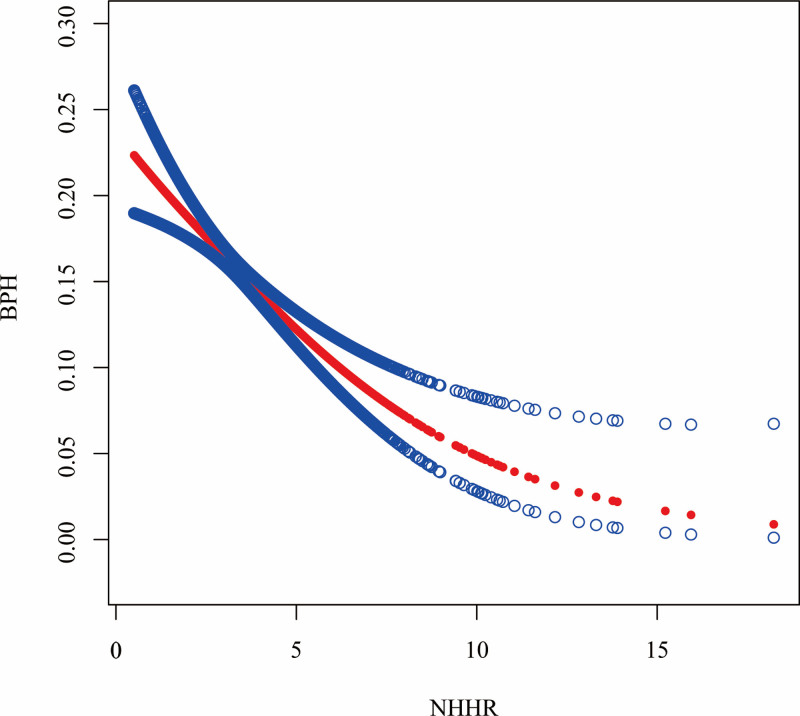
Correlation between NHHR and BPH. BPH = benign prostatic hyperplasia, NHHR = non-high-density lipoprotein cholesterol to high-density lipoprotein cholesterol.

We further investigated the nonlinear relationship between NHHR and BPH risk. By constructing a smooth curve, we found a nonlinear correlation was found between the occurrence of BPH and NHHR (Fig. 2). Using a biphasic linear model and a recursive algorithm, the study identified an inflection point for NHHR values at 3.00. In the regression model, no significant change in BPH risk was observed when the NHHR was below 3. When the NHHR was >3, the risk of BPH occurrence was reduced by 20% (95% CI = 0.72–0.88, *P *< .001) for each unit increase in NHHR (Table 4).

**Table 4:** Threshold effects between NHHR and BPH.

NHHR	Adjusted model	*P*-value
	OR (95%CI)	
<3	0.95 (0.79–1.15)	.639
≥3	0.80 (0.72–0.88)	<.001

95% CI = 95% confidence interval, BPH = benign prostatic hyperplasia, NHHR = non-high-density lipoprotein cholesterol to high-density lipoprotein cholesterol, OR = odds ratio.

### 3.4. Subgroup analysis

**Figure 3.**
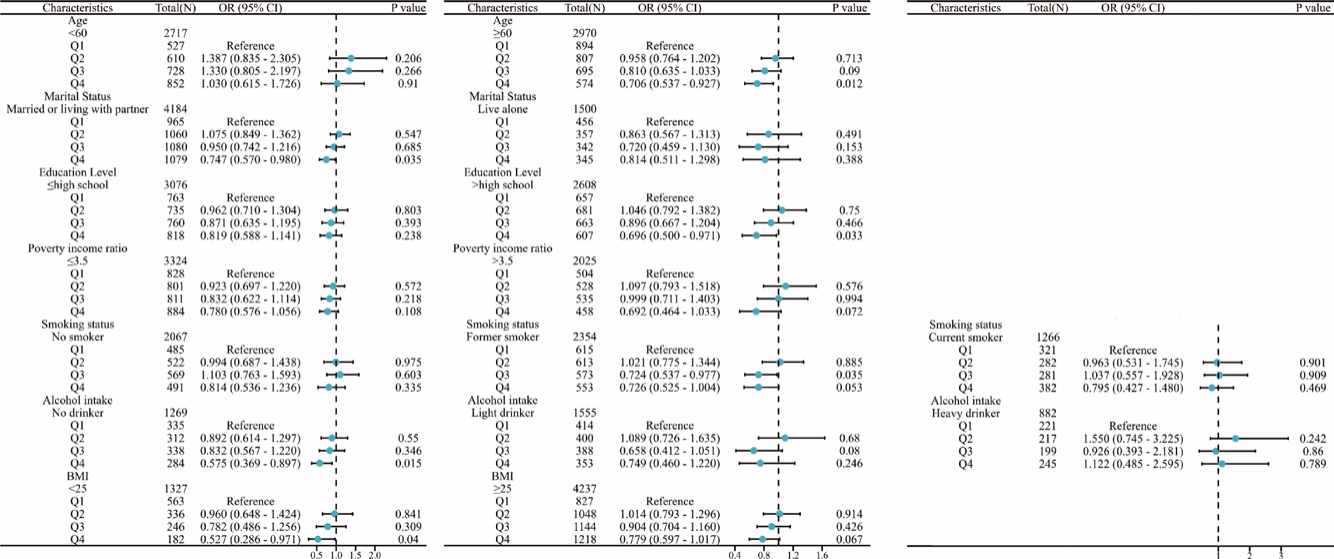
Subgroup analysis. Stratification adjustments were made for all variables (age, marital status, education level, PIR, smoking status, alcohol intake, and BMI) except for the stratification factor itself. BMI = body mass index, NHANES = National Health and Nutrition Examination Survey, PIR = poverty-to-income ratio.

Figure 3 shows the results of the subgroup analyses. Among those over 60 years of age, (OR_Q4vs1_ = 0.706, 95% CI = 0.537–0.927, *P *= .012), those married or living with partner (OR_Q4vs1_ = 0.747, 95% CI = 0.570–0.980, *P *= .035), individuals with a college degree (OR_Q4vs1_ = 0.696, 95% CI = 0.500–0.971, *P *= .033), former smokers (OR_Q3vs1_ = 0.724, 95% CI = 0.537–0.977, *P *= .035), no drinkers (OR_Q4vs1_ = 0.575, 95% CI = 0.369–0.897, *P *= .015) and those with BMI < 25 (OR_Q4vs1_ = 0.527, 95% CI = 0.286–0.971, *P *= .04). These findings suggest that the correlation between NHHR and BPH incidence may only exist in certain subgroups.

## 4. Discussion

The aim of this study was to investigate the relationship between NHHR and the incidence of BPH. There is still a gap in research on this issue. We analyzed 5687 participants. The results showed that BPH not only had lower TG, but also lower NHHR levels. The fourth quartile was negatively correlated with the risk of BPH compared to the first quartile of NHHR levels. In addition, this negative correlation remained in those over 60 years of age, married or living partner, college degree or higher, former smoker, no drinker, and BMI < 25. Further analysis by fitting smoothed curves and threshold effects showed a nonlinear relationship between NHHR and BPH, with a turning point at 3.00. When NHHR < 3, the increase in NHHR was not statistically associated with the risk of developing BPH. However, when NHHR > 3, the increase in NHHR was negatively associated with the risk of developing BPH.

Men over the age of 90 have a 90% chance of developing BPH. If patients are not treated promptly, it can lead to serious complications such as urinary retention and kidney failure.^[14,15]^ Several observational studies have shown a correlation between obesity and BPH. Specifically, BMI has a positive correlation with the occurrence of BPH.^[16]^ A prospective study further demonstrated an association between higher BMI, total body fat, abdominal fat levels, and an increased incidence of LUTS.^[17]^ Consumption of foods high in fat, such as butter and margarine, may increase the risk of BPH. This association may be attributed to inflammatory responses triggered by a high-fat diet, which may promote inflammation, hypoxia, and altered tissue structure in the prostate.^[18]^ A Mendelian randomization study suggests that a genetic predisposition to a larger waist circumference and prolonged sitting are independently and causally associated with an increased risk of BPH. Also, individuals genetically predisposed to a higher BMI may have a higher risk of BPH, which is supported by preliminary causal evidence. However, there is no clear evidence that genetic predisposition to relative carbohydrate, fat, protein, and sugar intake, smoking, and alcohol consumption is causally associated with the risk of BPH.^[19]^ The results of 1 study are striking in that it shows that increasing body fat content and LDL levels may play an important role in preventing the formation of BPH and LUTS.^[8]^ In conclusion, the role of LDL in the development of BPH remains unclear.

Non-high-density lipoprotein cholesterol to high-density lipoprotein cholesterol, the ratio of non-high-density lipoprotein cholesterol to high-density lipoprotein cholesterol, is an emerging lipid indicator and is regarded as an independent predictor of atherosclerosis. It plays a key role in predicting atherosclerotic plaque formation.^[13,20]^ Additionally, the diagnostic efficacy of NHHR in predicting diseases such as MS and insulin resistance has surpassed the traditional lipid indicators.^[21]^ Our study found that TC levels were significantly lower in patients with BPH compared to the normal population. More importantly, there was a negative correlation between NHHR levels and the risk of developing BPH. This finding may contradict conventional assumptions. One possible hypothesis is that LDL may play a protective role in the development of BPH. However, this does not imply that increasing LDL levels is advocated to reduce the risk of developing BPH, as LDL is recognized to increase the risk of developing cardiovascular disease. To further explore this relationship, subgroup analyses were conducted. It was found that the negative association between NHHR and the risk of BPH was mainly in the no drinker and BMI < 25 groups. We considered that this population has good lifestyle habits. This suggests that a moderate increase in NHHR may help prevent the occurrence of BPH in individuals with a normal BMI.

Metabolic syndrome (MetS) and BPH are usually co-morbidities, and chronic inflammation is the defining pathogenic factor in BPH. In MetS, dyslipidemia, in particular, is associated with the development of prostate inflammation. Thus, lipids may have deleterious effect on prostate cells and promote prostate inflammation, which is a key factor in the development and progression of BPH/LUTS.^[22]^ A meta-analysis identified HDL levels as the main determinant of BPH associated with MS.^[23]^ Specifically, HDL levels were negatively correlated with BPH.^[24]^ Our study found no significant difference in HDL levels in patients with BPH compared to the normal population. Importantly, NHHR levels were significantly lower in patients with BPH than in the normal population. Elevated NHHR levels appear to have a potential role in preventing the development of BPH. Therefore, the decision-making process for the use of cholesterol-lowering medications to alleviate BPH symptoms needs to be carefully considered by physicians. Although our study identified a possible association between NHHR and BPH risk, the limitations of the study cannot be ignored. First, the data used for the analysis were derived from an older dataset. Second, for reasons of the cross-sectional study design of the study, it was not possible to establish a direct causal relationship between NHHR and BPH. Finally, given that the data were derived from NHANES and the diagnosis of BPH relied on patient self-report, the detailed status of the disease remains to be clarified. To validate the findings of this study, further research is needed to explore the correlations between the various components of MS. Such studies would contribute to a more accurate understanding of how these components interact.

## 5. Conclusions

Non-high-density lipoprotein cholesterol to high-density lipoprotein cholesterol was negatively associated with the risk of developing BPH, suggesting that NHHR may be a potential protective factor for BPH. However, for reasons of the complexity of NHHR and BPH status, further prospective clinical trials are needed to confirm the potential role of lipids in BPH.

## Acknowledgments

We want to thank all participants in this study.

## Author contributions

**Conceptualization:** Mingtao Guo.

**Data curation:** Zhimin Lu.

**Formal analysis:** Zhimin Lu.

**Investigation:** Honglei Wan, Wenzhan Liu, Jiantong Zhao.

**Methodology:** Zhimin Lu, Wenzhan Liu, Jiantong Zhao.

**Project administration:** Mingtao Guo.

**Resources:** Honglei Wan, Jiantong Zhao.

**Software:** Honglei Wan, Wenzhan Liu.

**Supervision:** Mingtao Guo.

**Writing – original draft:** Mingtao Guo, Zhimin Lu.

**Writing – review & editing:** Mingtao Guo, Zhimin Lu.
